# The ORACLE Children Study: educational outcomes at 11 years of age following antenatal prescription of erythromycin or co-amoxiclav

**DOI:** 10.1136/archdischild-2015-310144

**Published:** 2016-08-11

**Authors:** Neil Marlow, Hannah Bower, David Jones, Peter Brocklehurst, Sara Kenyon, Katie Pike, David Taylor, Alison Salt

**Affiliations:** 1 Institute for Women's Health, University College London, London, UK; 2 Health Sciences Department, University of Leicester, Leicester, UK; 3 Institute of Applied Health Research, University of Birmingham, Edgbaston, UK; 4 Clinical Trials and Evaluation Unit, School of Clinical Sciences, University of Bristol, Bristol, UK; 5 Maternal and Newborn Health Initiative, International Federation of Gynaecology and Obstetrics, London, UK; 6 Great Ormond Street Hospital for Children and Institute of Child Health, University College London, London, UK

**Keywords:** School Health, Fetal Medicine, Neurodevelopment, Outcomes research

## Abstract

**Background:**

Antibiotics used for women in spontaneous preterm labour without overt infection, in contrast to those with preterm rupture of membranes, are associated with altered functional outcomes in their children.

**Methods:**

From the National Pupil Database, we used Key Stage 2 scores, national test scores in school year 6 at 11 years of age, to explore the hypothesis that erythromycin and co-amoxiclav were associated with poorer educational outcomes within the ORACLE Children Study.

**Results:**

Anonymised scores for 97% of surviving children born to mothers recruited to ORACLE and resident in England were analysed against treatment group adjusting for key available socio-demographic potential confounders. No association with crude or with adjusted scores for English, mathematics or science was observed by maternal antibiotic group in either women with preterm rupture of membranes or spontaneous preterm labour with intact membranes. While the proportion receiving special educational needs was similar in each group (range 31.6–34.4%), it was higher than the national rate of 19%.

**Conclusions:**

Despite evidence that antibiotics are associated with increased functional impairment at 7 years, educational test scores and special needs at 11 years of age show no differences between trial groups.

**Trial registration number:**

ISCRT Number 52995660 (original ORACLE trial number).

**What is already known on this topic?**

- Antibiotics given to women with preterm rupture of membranes and no overt infection have neonatal benefit and appear safe in terms of childhood outcomes at 7 years of age.
- In contrast, when administered to women with spontaneous preterm labour with intact membranes there is no neonatal benefit and evidence of poorer outcomes in terms of neurodevelopmental impairment and cerebral palsy at 7 years of age.
- Previous studies are open to ascertainment bias in outcomes in middle childhood.

**What this study adds?**

- Using independently collected and scored national attainment tests of English, mathematics and science, we demonstrate no differences in long-term educational outcomes at 11 years, or in special needs, following antenatal prescription of antibiotics.
- While attainment test results are within national norms, special needs requirements among these children are higher.
- The use of anonymised educational national data provides good coverage of the population and a robust middle childhood outcome.

## Introduction

The ORACLE Children Study evaluated the outcome of children born to women who were entered into the ORACLE study and were randomly assigned to receive erythromycin, co-amoxiclav or placebo in two factorial trials relating to women in spontaneous labour with intact membranes (SPL)^1^ or with prelabour preterm rupture of membranes (PROM).^2^ Children whose mothers received antibiotics during SPL had an excess of impairments at 7 years, in contrast to those whose mothers were entered into the PROM arm of the trial. These data, however, are based on follow-up of 71% (SPL) and 75% (PROM), respectively, of the children born to mothers who took part.

Reports of long-term outcomes following perinatal trials aim for as complete ascertainment as is possible. One potential solution to this is to use nationally collected educational data. We have recently reported outcomes for 7-year-old children using Key Stage 1 (KS1) results.^3^ The disadvantage of these tests is that they represent the performance of the child based on teacher assessment and do not include pupils in all schools. In contrast, Key Stage 2 tests (KS2) are universal, standardised, scored independently and reported unmodified; they thus provide an opportunity to review an independently collected and scored measure of children's educational attainment outcomes in relation to trial groups.

This paper examines the hypothesis that, within the ORACLE trial, treatment of women in SPL or with PROM leads to changes in brain development that are manifested as altered educational attainment at KS2 in their children.

## Methods

### The ORACLE study

The original trial recruited 4221 and 4148 women, respectively, to the SPL and PROM trials. Questionnaire-based follow-up information was obtained for 3196 (71%)^1^ and 3298 of 4358 children (75%) for SPL and PROM arms of the study,^2^ respectively. Mean gestational age at birth was 38 and 34 weeks, respectively, for SPL and PROM arms of the study. Overall, 6295 of 8369 (75%) children were resident in England at the completion of the ORACLE Children Study and formed the population for this study.

### Data

The Department for Education (DfE) supplied full KS2 data at 11 years of age anonymously for ORACLE children categorised by treatment group. Linkage was performed by the DfE, and based on information we held on individual children. Matching was carried out probabilistically based on forename, surname, date of birth and postcode. No further information on those not matched is available because of data anonymity requirements. The KS2 data supplied comprised continuous scores for English, mathematics and science and a DfE measure of educational progress entitled contextual value added (CVA). CVA measures the progress made by pupils from the end of KS1 to the end of KS2, adjusting for external factors, such as the child's sex, changing school and levels of deprivation, that impact pupil results at school. For further information on the derivation of this measure, see http://www.education.gov.uk/schools/performance/archive/schools_10/cvacalc.pdf (accessed 30 March 2016). In addition, the data set included the following data: age at the beginning of the KS2 assessment year, ethnicity of the child, English as first language (yes/no), free school meal eligibility (yes/no), child's sex, Income Deprivation Affecting Children Index (IDACI) score,^4^ school type (community, voluntary/faith, foundation, other) and academic year of KS2 assessment, in addition to ORACLE group allocation information.

The DfE Research Unit was sent a computer file of study members' records in four groups (according to their treatment allocation), and data were returned with identifiers removed (ie, anonymised) separately for each of the four treatment groups. Identifiers comprised either the name, date of birth and postcode for each child or the Unique Pupil Identifier (UPI) for linking to KS2 outcomes; individual identifiers were then removed (leaving only the trial allocation) on the file when returned to the authors. As this was an anonymised analysis, no research ethics committee permission was required. Permission was obtained from the DfE Research Unit to use these data.

### Statistical analysis

KS2 data were dichotomised into level 4 or above (considered to be appropriate progress at school) and below level 4, and Mantel–Haenszel ORs were calculated comparing erythromycin and co-amoxiclav separately for effects on the odds of achieving less than a level 4 at KS2.

Log-linear models including covariates for erythromycin and co-amoxiclav were fitted for the aggregate KS2 levels (<2, 2, 3, 4, 5 and 6) for English, mathematics and science. Linear regression models were fitted for the raw scores for each of the three KS2 subjects. All models are adjusted for a selection of covariates. Covariates considered for inclusion within log-linear and linear regression models in addition to the antibiotic indicators were: age at the beginning of the KS2 assessment year, ethnicity, English as first language, free school meal eligibility, sex, IDACI score, school type and KS2 assessment year.

Univariate analyses were performed first to determine which covariates should be considered for inclusion within the multivariate models. All models included erythromycin and co-amoxiclav terms. Covariates (including interaction terms between the two antibiotic indicators as in the original analyses^1^) were selected through the use of likelihood ratio tests. All statistical modelling including model selection was performed for the PROM and SPL cohorts separately. Due to large numbers of missing values for some variables (see online supplementary table S2), complete case analyses and analyses which considered only variables with <10% missing data were performed (see online supplementary tables S3 and S4).

10.1136/fetalneonatal-2015-310144.supp1 Supplementary tables

## Results

Within the ORACLE Study, for children who were born to women resident in England at the conclusion of the follow-up study (2007), anonymised KS2 data were available for up to 6087 of 6295 children recruited to the original trial (97%). The DfE-supplied baseline characteristics of children born to women in the SPL and PROM groups appear similar across all variables (see online supplementary table S1).

Analysis of continuous scores and antibiotic effects was carried out separately for women in PROM and SPL groups and reported in their four factorial groups (table 1). Attainment scores in English, maths and science were similar for those who received erythromycin or not, and for those who received co-amoxiclav or not; scores between the four groups did not vary, with similar IQRs for each. The CVA score showed evidence of a small positive improvement in performance across KS1 to KS2, which did not vary by antibiotic group for either PROM or SPL. Special needs were identified in similar proportions in each group/condition ranging from 31.6% to 34.4%.

**Table 1:** Summary statistics for contextual value added score and raw scores for English, maths and science by ORACLE treatment groups

	Erythromycin	No erythromycin	Co-amoxiclav	No co-amoxiclav
**Preterm rupture of membranes**				
	1512	1528	1523	1517
English (n)	1275	1304	1303	1276
Median (IQR)	57 (45 to 67)	58 (46 to 68)	58 (46 to 68)	57 (45 to 67)
Mathematics (n)	1265	1314	1300	1279
Median (IQR)	64 (47 to 78)	64 (47 to 79)	64 (47 to 78)	64 (47 to 79)
Science (n)	798	814	812	800
Median (IQR)	59 (49 to 67)	59 (50 to 67)	60 (50 to 67)	58 (49 to 67)
Contextual VA (n)*	1090	1113	1105	1098
Median (IQR)	100.1 (98.6 to 101.5)	100.1 (98.7 to 101.7)	100.1 (98.6 to 101.5)	100.2 (98.7 to 101.6)
Special needs (n)†	1356	1387	1379	442/1364
n (%)	467 (34.4%)	438 (31.6%)	463 (33.6%)	(32.4%)
**Spontaneous preterm labour**				
**Total with data**	**1550**	**1497**	**1498**	**1549**
English (n)	1328	1285	1275	1338
Median (IQR)	56 (45 to 66)	56 (45 to 67)	57 (45 to 68)	56 (44 to 66)
Mathematics (n)	1334	1287	1281	1340
Median (IQR)	62 (46 to 77)	62 (47 to 77)	63 (47 to 78)	61 (46 to 77)
Science (n)	813	787	776	824
Median (IQR)	59 (48 to 66)	58 (48 to 66)	59 (48 to 67)	58 (48 to 66)
Contextual VA (n)*	1138	1066	1065	1139
Median (IQR)	100.0 (98.5 to 101.6)	100.0 (98.5 to 101.5)	101.1 (98.7 to 101.6)	100.0 (98.3 to 101.5)
Special needs (n)†	1421	1345	1353	1413
n (%)	475 (33.4%)	426 (31.7%)	427 (31.6%)	474 (33.5%)

Scores are the number of marks achieved.

*Contextual value added (VA) scores are expressed as a per cent improvement compared with previous assessments; for further definitions, see http://www.education.gov.uk/schools/performance/archive/schools_10/s3.shtml; accessed 30 March 2016.

†Special need categories: school action, school action plus (for 2004–2006 also includes educational statements and statutory assessment); for definitions, see, https://http://www.gov.uk/children-with-special-educational-needs/support-before-september-2014; accessed 30 March 2016.

Attainment of KS2 level 4 is considered appropriate progress within this assessment paradigm; we dichotomised KS2 results and present them for PROM and SPL populations (table 2). ORs for each subject did not vary significantly by antibiotic group in either population. We then derived a log-linear model for aggregated KS2 levels adjusted for baseline data, including only variables with <10% missing data, shown in table 3. Again no effect of antibiotic or maternal trial population was observed. Finally, we performed a linear regression using the crude KS2 scores, adjusting as before, and found no significant variation of effect size by antibiotic or trial group (table 4).

**Table 2:** Educational attainment lower than level 4* in English, mathematics and science at Key Stage 2 by ORACLE treatment groups

	Erythromycin	No erythromycin	Co-amoxiclav	No co-amoxiclav
**Preterm rupture of membranes**				
	1512	1528	1523	1517
English (n)				
<Level 4 (%)	350 (25.6)	342 (24.6)	326 (23.5)	366 (26.7)
OR (95% CI)†	1.05 (0.89 to 1.26)		0.85 (0.71 to 1.01)	
Mathematics (n)				
<Level 4 (%)	377 (27.7)	364 (26.1)	366 (26.4)	375 (27.4)
OR (95% CI)	1.08 (0.91 to 1.29)		0.95 (0.80 to 1.13)	
Science (n)				
<Level 4 (%)	126 (15.1)	129 (15.3)	119 (14.1)	136 (16.3)
OR (95% CI)	0.98 (0.75 to 1.30)		0.84 (0.64 to 1.11)	
**Spontaneous preterm labour**				
	**1550**	**1497**	**1498**	**1549**
English (n)				
<Level 4 (%)	352 (24.8)	335 (24.8)	321 (23.8)	366 (25.8)
OR (95% CI)	1.00 (0.84 to 1.19)		0.90 (0.75 to 1.07)	
Mathematics (n)				
<Level 4 (%)	394 (27.7)	360 (26.6)	354 (26.1)	400 (28.2)
OR (95% CI)	1.06 (0.89 to 1.25)		0.90 (0.76 to 1.07)	
Science (n)				
<Level 4 (%)	130 (15.4)	117 (14.5)	105 (13.2)	142 (16.7)
OR (95% CI)	1.07 (0.81 to 1.42)		0.76 (0.57 to 1.01)	

*Level 4 is the minimum standard which children are expected to achieve.

†OR, Mantel–Haenszel ORs (95% CIs).

**Table 3:** Erythromycin and co-amoxiclav HRs (95% CI) from the adjusted log-linear models for aggregate KS2 levels*

	PROM		SPL	
	Erythromycin	Co-amoxiclav	Erythromycin	Co-amoxiclav
*English*				
CC	1.00 (0.96 to 1.05)	1.03 (0.99 to 1.08)	0.97 (0.92 to 1.03)	1.01 (0.95 to 1.06)
<10%	1.00 (0.96 to 1.03)	1.02 (0.98 to 1.06)	0.98 (0.95 to 1.02)	1.01 (0.98 to 1.05)
*Mathematics*				
CC	0.98 (0.94 to 1.03)	1.02 (0.97 to 1.07)	0.99 (0.95 to 1.03)	1.00 (0.96 to 1.05)
<10%	0.98 (0.95 to 1.02)	1.00 (0.96 to 1.04)	0.99 (0.95 to 1.02)	1.01 (0.97 to 1.05)
*Science*				
CC	1.00 (0.95 to 1.05)	1.02 (0.97 to 1.07)	0.98 (0.93 to 1.04)	1.01 (0.96 to 1.07)
<10%	1.00 (0.95 to 1.04)	1.01 (0.97 to 1.06)	0.99 (0.94 to 1.04)	1.02 (0.97 to 1.07)

*See supplemental table 2 for details of covariates used (with <10% missing data).

<10%, <10% missing data; CC, complete case analysis; KS2, Key Stage 2; PROM, preterm rupture of membranes; SPL, spontaneous labour with intact membranes.

**Table 4:** Erythromycin and co-amoxiclav estimates (95% CI) from the adjusted linear regression models for raw KS2 scores*

	PROM		SPL	
	Erythromycin	Co-amoxiclav	Erythromycin	Co-amoxiclav
*English*				
CC	−0.54 (−2.05 to 0.97)	1.03 (−0.48 to 2.54)	0.18 (−1.46 to 1.82)	0.79 (−0.86 to 2.43)
<10%	−1.05 (−2.29 to 0.19)	−0.71 (−0.54 to 1.95)	−0.42 (−1.61 to 0.77)	1.02 (−0.18 to 2.21)
*Mathematics*				
CC	−1.10 (−3.18 to 0.97)	0.82 (−1.26 to 2.89)	0.18 (−2.07 to 2.42)	1.46 (−0.80 to 3.71)
<10%	−0.74 (−2.40 to 0.92)	0.09 (−1.57 to 1.74)	−0.07 (−1.67 to 1.54)	1.55 (−0.05 to 3.15)
*Science*				
CC	0.17 (−1.34 to 1.67)	1.64 (0.14 to 3.15)	1.47 (−0.40 to 3.34)	1.31 (−0.57 to 3.18)
<10%	−0.07 (−1.34 to 1.19)	0.94 (−0.33 to 2.20)	0.37 (−0.91 to 1.65)	0.96 (−0.32 to 2.24)

*See supplemental table 3 for details of covariates used (with <10% missing data).

<10%, <10% missing data; CC, complete case analysis; KS2, Key Stage 2; PROM, preterm rupture of membranes; SPL, spontaneous labour with intact membranes.

## Discussion

Educational attainment is a good marker for cognitive outcome within perinatal cohorts, and the prevalence of special needs is closely related to gestational age at birth.^5^ We have used the results of a national, independently administered and scored educational test battery to evaluate outcome for children of women recruited to the ORACLE studies.^6^ ^7^ No effect of trial intervention has been demonstrated on continuous outcome scores, a measure of education progress (CVA measure), or on criterion-referenced attainment or on identified special needs. Adjusted models yield very similar results to the unadjusted analyses here, although it should be noted that adjustment for factors postrandomisation in principle runs the risk of over adjustment. The data show similarities to national data—in 2010, 80% of children in England at KS2 achieved level 4 or better (the national ‘floor’ for test results) in English and 79% in mathematics (DfE School performance tables (see http://www.education.gov.uk; accessed 31 July 2014)). In ORACLE, respective figures were 75% and 74%. Nationally, 19% of 11-year-old pupils have special needs, compared with 31–34% across all study groups. It should be remembered that a high proportion of the ORACLE trial participants gave birth prematurely (36% in SPL and 85% in PROM groups, respectively) and one would expect lower attainment and higher rates of special needs in ex-preterm children.^5^

The strength of using national data lies in their independent ascertainment, coverage and low dropout rate (4% of children). The tests may vary slightly from year to year, but they are continually restandardised between years to produce consistent outcome data. The advantage of near complete coverage from this source, as against 71–75% from parent-based follow-up in the original ORACLE Children Study,^1^ ^2^ is in the avoidance of ascertainment bias.

Within a trial of antenatal interventions aimed at improving neonatal and childhood condition, national educational data provide a sensitive and robust measure of outcome. However, the limitation of using anonymised data lies in our inability to associate data from education with our research database without individual parental permission; hence, the effect of linkage information and missing data (which for some results means we only have information on half of the cohort, eg, in Science), and thus the degree of bias contributed thereby, cannot be ascertained, more granular evaluation of the effects of the two antibiotics in the two trial groups is not possible, and the influence on educational attainment of other perinatal events or disability ratings from the main study cannot be determined. Furthermore, current data protection strategies on the part of the DfE and the researchers may make a study such as this difficult in the future by insisting on individual participant permissions.

Within the original ORACLE Children Study reports, we raised concern about a small but significant increase in the risk of cerebral palsy, following antibiotic prescription in spontaneous preterm labour. Subsequently, the use of macrolide antibiotics in term pregnancy has been associated with the occurrence of cerebral palsy using the Health Improvement Network (adjusted HR: 1.78, 95% CI 1.18 to 2.69),^8^ which appears to confirm our concerns. Cerebral palsy is a rare outcome in the population, and within the ORACLE studies, it comprises <3% of the population.^9^ Cerebral palsy may be associated with learning problems but for children in this study the functional motor deficit associated with cerebral palsy on the ORACLE Children Study tended to be less severe,^9^ and anticipated effects on academic attainment would also be less severe. Furthermore, the later the age at follow-up will allow the effect of social and environmental influences to dilute the important neonatal influences. For example, despite the success of neonatal caffeine in improving neonatal and outcomes in the second year, including cerebral palsy rates, re-evaluation of the population at 5 years failed to demonstrate continuing benefit.^10^ Such findings provide reassurance of longer term safety but do not diminish identified short-term influences.^11^ Thus, any effect on educational scores from this excess would be overwhelmed by the results of the remainder of the population without cerebral palsy, but it is reassuring to see no evidence of poorer educational attainment overall.

The ORACLE study has demonstrated short-term neonatal benefit from the use of antibiotics for women with preterm rupture of the membranes and suggested longer term harm from giving women antibiotics in the presence of SPL. The follow-up of those children at 7 years of age whose mothers had SPL suggested that the prescription of erythromycin (with or without co-amoxiclav) was associated with an increase in the proportions of children with any level of functional impairment and cerebral palsy. This paper provides reassurance from the use of standardised national attainment tests and special needs for 6087 children resident in England that no benefit or harm was demonstrated on overall educational attainment for these children at 11 years.
