# Supplementary material for: The ORACLE Children Study: educational outcomes at 11 years of age following antenatal prescription of erythromycin or co-amoxiclav
Source: Arch Dis Child Fetal Neonatal Ed. 2016 Aug 11;102(2):F131–5. doi: 10.1136/archdischild-2015-310144 (PMC5339554; doi:10.1136/archdischild-2015-310144)
Supplement: Supplementary tables [file fetalneonatal-2015-310144supp_tables.pdf]

The ORACLE Children Study: Educational outcomes at 11 years of age following antenatal prescription of erythromycin or co-amoxiclav

APPENDIX – supplemental tables

Table s1: Baseline characteristics of those participants for whom KS2 data were returned by antibiotic group

	**Erythromycin**<br><br>**N=3025**<br>n(%)	**No erythromycin**<br>**N=3062**<br>n(%)	**Co-amoxiclav**<br><br>**N=3066**<br>n(%)	**No co-amoxiclav**<br>**N=3021**<br>n(%)
**Age**	3025 (100)	3062 (100)	3066 (100)	3021 (100)
years, mean (SD)	10.5 (0.29)	10.5 (0.28)	10.5 (0.28)	10.5 (0.28)
**Academic year**	3025 (100)	3062 (100)	3066 (100)	3021 (100)
2004/2005	13 (0.4)	12 (0.4)	12 (0.4)	13 (0.4)
2005/2006	175 (5.8)	189 (6.2)	180 (5.9)	184 (6.1)
2006/2007	320 (10.6)	343 (11.2)	330 (10.8)	333 (11.0)
2007/2008	465 (15.4)	446 (14.6)	456 (14.9)	455 (15.1)
2008/2009	693 (22.9)	698 (22.8)	723 (23.6)	668 (22.1)
2009/2010	825 (27.3)	839 (27.4)	839 (27.4)	825 (27.3)
2010/2011	534 (17.7)	535 (17.5)	526 (17.2)	543 (18.0)
**First language**	2544 (84.1)	2577 (84.2)	2585 (84.3)	2536 (83.9)
English	2449 (96.3)	2494 (96.8)	2499 (96.7)	2444 (96.4)
**Free school meal eligibility**	2231 (73.8)	2239 (73.1)	2263 (73.8)	2207 (73.1)
Eligible	553 (24.8)	533 (24.5)	611 (27.0)	567 (25.7)
**Ethnicity**	2048 (67.7)	2068 (67.5)	2091 (68.2)	2025 (67.0)
White	1791 (87.4)	1820 (88.0)	1844 (88.2)	1767 (87.3)
**Gender**	3025 (100)	3062 (100)	3066 (100)	3021 (100)
Male	1604 (53.0)	1640 (53.6)	1659 (54.1)	1585 (52.5)
**IDACI score**	1782 (58.9)	1820 (59.4)	1827 (59.6)	1775 (58.8)
mean(SD)	0.25 (0.19)	0.26 (0.19)	0.25 (0.19)	0.26 (0.20)
**School type**	3023 (99.9)	3061 (99.9)	3064 (99.9)	3020 (99.9)
Community	2131 (70.5)	2100 (68.6)	2125 (69.4)	2106 (69.7)
Voluntary/faith	791 (26.2)	859 (28.1)	842 (27.5)	808 (26.8)
Foundation	96 (3.2)	90 (2.9)	88 (2.9)	98 (3.3)
Other	5 (0.2)	12 (0.4)	9 (0.3)	8 (0.3)
**Educational needs**	2732 (90.3)	2777 (90.7)	2777 (90.6)	2732 (90.4)
Special needs (SEN)	864 (31.6)	942 (33.9)	916 (33.0)	890 (32.6)

Key: IDACI: income deprivation affecting children index

Table s2: Proportion of missing data for the potential covariates for the Prelabour preterm rupture of membranes (PROM) and spontaneous labour (SPL) cohorts

Variable	PROM	SPL
**Academic year**	0	0
**Age**	0	0
**Ethnicity**	33.2%	31.6%
**First language**	16.9%	14.9%
**Free school meal eligibility**	28.4%	24.8%
**Gender**	0	0
**IDACI**	33.8%	47.8%
**School type**	1.0%	0

Key: IDACI: income deprivation affecting children index

Table s3: Covariates included in log-linear models for the PROM and SPL cohorts when conducting a complete case analysis and when only considering variables with <10% missing.

	**PROM**		**SPL**	
	**Complete case**	**<10% missing**	**Complete case**	**<10% missing**
**English**	FSM<br>Gender<br>Age	Age<br>Gender	FSM<br>Gender<br>IDACI	Gender
**Mathematics**	FSM<br>IDACI<br>Age	Age	FSM	School
**Science**	IDACI<br>Age	Age	FSM	

Key: FSM: Free school meals; IDACI: income deprivation affecting children index

Table s4: Covariates included in linear regression models for the PROM and SPL cohorts when conducting a complete case analysis and when only considering variables with <10% missing.

	**PROM**		**SPL**	
	**Complete case**	**<10% missing**	**Complete case**	**<10% missing**
**English**	Age<br>FSM<br>Gender<br>IDACI	Age<br>Gender<br>School	Age<br>FSM<br>Gender<br>IDACI	Age<br>Gender<br>School
**Mathematics**	Age<br>FSM<br>Gender<br>IDACI	Age<br>Gender<br>School	Age<br>FSM<br>IDACI	Academic year<br>Age<br>Gender<br>School
**Science**	Age<br>EFL<br>FSM<br>IDACI	Age<br>School	FSM<br>IDACI	Age

Key: FSM: Free school meals; IDACI: income deprivation affecting children index; EFL: English as first language
